# How adherens junctions move cells during collective migration

**DOI:** 10.12703/r/10-56

**Published:** 2021-06-15

**Authors:** Shafali Gupta, Alpha S Yap

**Affiliations:** 1 Division of Cell and Developmental Biology, Institute for Molecular Bioscience, The University of Queensland, St. Lucia, Brisbane, Queensland, Australia 4072

**Keywords:** Collective cell migration, epithelia, adherens junctions, cadherins, cytoskeleton, intercalation

## Abstract

In this review, we consider how the association between adherens junctions and the actomyosin cytoskeleton influences collective cell movement. We focus on recent findings which reveal different ways for adherens junctions to promote the locomotion of cells within tissues: through lamellipodia and junctional contraction. These contributions reflect how classic cadherins establish sites of cortical actin assembly and how adherens junctions couple to contractile actomyosin, respectively. The diverse interplay between cadherin adhesion and the cytoskeleton thus provides different ways for adherens junctions to support epithelial locomotion.

## Introduction

**Figure 1.**
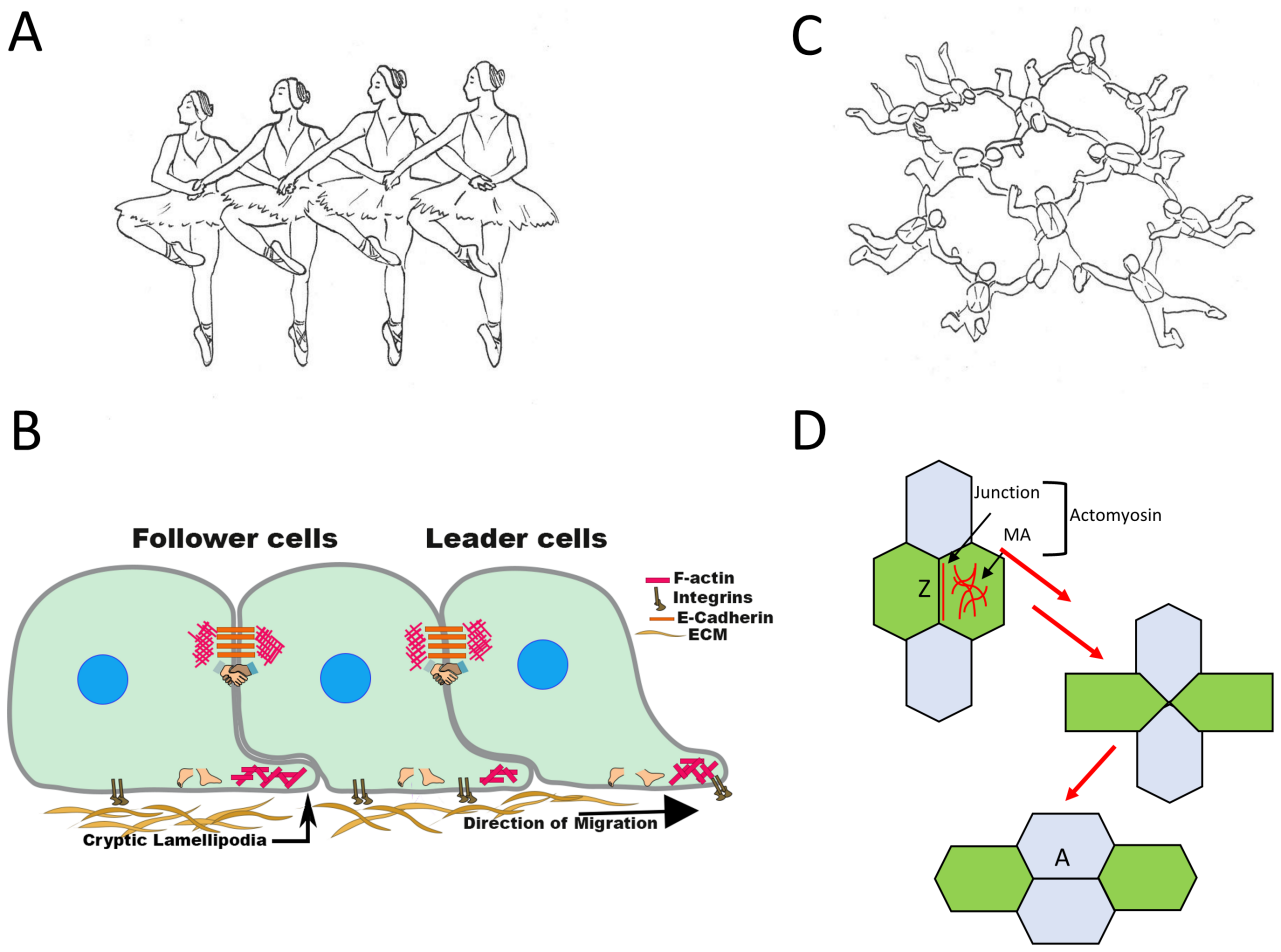
Different locomotor apparatuses in collective cell migration and different roles for adherens junctions (AJs) in collective cell migration. Like the corps de ballet (**A**), cells may principally move by translocating on their extracellular matrix (ECM) (through lamellipodial protrusions, cortical flows, and tractions) but retain cell–cell cohesion by cell–cell adhesion (**B**). Like formation skydivers (**C**), cells may exert forces on one another through AJs for morphogenetic cell rearrangement (**D**). This is illustrated by the T1 transition where vertical junction “Z” shrinks, yielding a transient four-way vertex. This is resolved by the creation of a horizontal junction “A”. Junction shrinkage involves contraction by medial-apical (MA) or junctional pools of actomyosin or both.

Collective cell migration can take many different forms. This diversity is evident if we consider the movement of physically coherent tissues, such as epithelia and endothelia. One mode of epithelial migration is seen during wound healing or when enterocytes migrate up the crypt–villus axis of the intestine^1–4^. Here, cells are thought to principally locomote by crawling upon their underlying extracellular matrix (ECM) while retaining physical contact with one another. One could liken this mode of migration to a corps de ballet, where the legs of the dancers drive their movement but they also link arms to stay together (Figure 1A and B). In a second form of epithelial movement, contraction of cell–cell junctions generates the locomotor force for tissue movement^5–8^. Characteristically, these tissue movements involve rearrangement of the constituent cells, as is seen during convergent extension^9–11^. This second class of epithelial movement is like groups of skydivers who (re)arrange by pulling on their joined arms (Figure 1C and D). Reality, of course, can add even more complexity by combining protrusive crawling with junctional contractility^11,12^.

Classic cadherin adhesion molecules play important roles in collective migration. However, given the underlying diversity of these different forms of “collective” movement, it is perhaps not surprising to find that cadherins have been reported to have divergent effects in different forms of collective migration. For example, depleting cadherin increased the movement of cells within confluent epithelial monolayers^13^ or in the early Zebrafish embryo^8^. In contrast, inhibiting cadherin adhesion disrupted the collective movements of cells during convergent extension in the gastrulating frog embryo^14^ and also perturbed Schwann cell migration in wound-healing assays^15^. One possibility is that cadherins contribute to multiple processes which impinge on collective migration. The extent to which these contributions vary in different biological situations would then affect the overall impact of cadherins.

In this review, we will focus on new evidence that implicates cadherin-based adherens junctions (AJs) in the locomotor mechanisms of collective migration. Much is now known about the machinery that individual cells use to translocate within the complex environment of the body. Common features in this mechanistic diversity are cytoskeleton-based processes that allow cells to (a) protrude and (b) exert mechanical forces to translocate their centre of mass. It now appears that cells have found inventive ways to harness the dynamic cytoskeleton of AJs to achieve these purposes: through (1) ECM-based lamellipodia and (2) the contraction of junctions themselves. Many of these studies have been performed in epithelia, but we also discuss experiments performed in other tissues that use classic cadherins for cohesion.

## Brief notes on the cortical cytoskeleton at adherens junctions

It has been known for many years that cadherin adhesion functions in close partnership with the actin cytoskeleton. We have often visualised that partnership as a “core” cadherin molecular complex (reviewed in 16). Here, the type 1 cadherin transmembrane receptor associates with actin filaments via its intermediary catenin proteins: specifically, α-catenin binds F-actin directly and is, in turn, coupled to the cadherin via β-catenin. This model has guided research in the molecular mechanisms of cadherins for over 30 years and continues to yield fundamental insights. Only now, for instance, with the application of cryoelectron microscopy, has the molecular interaction of α-catenin and F-actin been able to be visualised at high resolution^17,18^.

However, the model of a “core” complex is also incomplete. We now appreciate that a diverse range of cytoskeletal elements are recruited to interact with the cadherin adhesion system^19^. These interactions are often likely to be transient, substoichometric, and indirect. Indeed, super-resolution optical imaging indicates that cadherin adhesions can be understood as nano-scale structures where many different cytoskeletal proteins are organised in layers at the junctional cortex^20^. Thus, cadherins and their associated actomyosin cortex can be considered a dynamic membrane-spanning composite that has great biological and mechanical versatility. This “expanded” understanding of cadherins and their cortex has been reviewed in detail elsewhere^21^. For the purposes of this discussion, we would highlight the following features.

First, the cadherin-associated cytoskeleton can display different functional states, ranging from active assembly of filament networks to contractile actomyosin networks. This reflects the cortical action of multiple signal transduction pathways and actin regulatory proteins, many of which can be recruited to the junctional cortex in response to cadherin adhesion. Key signals include the classic actin regulatory small GTPases, Rac and RhoA, and a plethora of actin-binding proteins that have distinct effects on actin filament dynamics and organisation. At a first approximation, these effectors can be thought to promote protrusive movements (Rac, Arp2/3 actin nucleator) or cell contractility (RhoA, formins).

Second, the junctional cytoskeleton can exert different mechanical effects, depending on its activity state. These include the generation of potentially protrusive forces by actin assembly^22,23^, application of contractile tension to the junction^5–7,24^, and regulating the mechanical stiffness of the junction itself^25^. These properties in turn determine features such as junctional length (e.g., shortened by increasing contractile line tension in the junction) that, as we shall see, can influence cell movement. The junctional cytoskeleton thus has the capacity to function as a versatile biomechanical tool during collective migration.

## Lamellipodia, cadherin junctions, and epithelial migration

Lamellipodia are a common mode of protrusion that cells use when they crawl upon extracellular matrices. Historically, lamellipodia have been most readily visualised when epithelial and endothelial migration is studied in wound-healing assays (Figure 1B). Here, migration begins with the extension and translocation of cells found at the margin of the “wound”, the so-called leader cells. This is eventually accompanied by the movement of cells behind them (also known as “follower” cells). Leader cells are also evident in three-dimensional models of collective migration, such as epithelial and endothelial tubulogenesis^26,27^, where they are thought to play important roles in controlling the timing and direction of migration.

Prominent lamellipodia often appear in these assays as the first step in leader cell migration. This prominence has led to the notion that leader cells may be dominant drivers of locomotion in these moving sheets of cells. Furthermore, studies with traction force microscopy have reported that leader cells could generate forces large enough to be capable of dragging follower cells along^4^. This suggested that the leaders may be the dominant drivers of locomotion in collective migration. In the extreme case, actively migrating leader cells could pull passive follower cells along with them and AJs would provide the cell–cell adhesion that was necessary for followers to be pulled along.

However, in other biological circumstances, collective migration occurs without evident leader cells. This is exemplified by the small intestine, where epithelial cells move constantly up from the crypts until they are shed by extrusion at the villus tips^1^. Here, there is no evident “edge” to the migrating population, nor obvious leader cells. Also, leader cells can exchange places with follower cells, suggesting that their fate is not predetermined^27^. Moreover, leader cells are often found at the tips of structures that can be much longer (millimetres) than the length of a cell, as is the case for mammary ducts and developing vessels. It seems unlikely that pulling forces from a limited number of tip cells can be transmitted over such length scales. These examples suggest that collective migration may not be driven simply by the dominant action of leader cells.

Indeed, it is increasingly evident that follower cells themselves are actively migratory. In particular, so-called cryptic lamellipodia are generated by follower cells^1,28–30^. Like other kinds of lamellipodia, these cryptic lamellipodia are distinguished by actin assembly at their leading edges which is mediated by the Arp2/3 complex and the WAVE regulatory complex which activates Arp2/3^1,30^. This mode of branched actin assembly contributes to locomotility by generating protrusive forces for lamellipodial extension and also by contributing to retrograde flow of the actin cortex^31^. The presence of cryptic lamellipodia then suggested that follower cells may be actively propulsive and contribute to collective migration.

Recently, that hypothesis was tested directly. To do this, Ozawa *et al*. (2020) inhibited lamellipodia formation by depleting epithelial cells of either p34 (a component of the Arp2/3 complex) or Nap1 (part of the WAVE regulatory complex) by RNA interference (RNAi) (knockdown, or KD)^30^. The authors mixed these cells with wild-type cells so that cultures contained clusters of KD cells surrounded by wild-type cells and then examined how these subpopulations behaved in wound-healing assays. Epithelial integrity was not overtly compromised, but both p34 KD and Nap1 KD cells were left behind as these mixed monolayers migrated, such that the leading front consisted largely of wild-type cells. In contrast, cells expressing control small interfering RNAs (siRNAs) were readily found with wild-type cells at the leading fronts^30^. Therefore, disrupting lamellipodia appeared to compromise the ability of follower cells to keep up with the rest of the moving population of cells. This indicated that cryptic lamellipodia were important for collective migration and implied that self-propulsion might be a distinguishing feature of cells within the migrating population.

Ozawa *et al*. also found a surprising link between AJs and cryptic lamellipodia. Live-cell imaging revealed that the dynamic actin networks at the leading edges of cryptic lamellipodia appeared to derive from the cortical cytoskeleton of AJs. Here, it is useful to note that AJs themselves are sites of branched actin filament assembly that is mediated by Arp2/3 and the WAVE regulatory complex^32–34^, the same nucleator apparatus used in lamellipodia. One possibility is that lamellipodia and AJs compete for the services of the WAVE-Arp2/3 actin nucleator. However, Ozawa *et al*. also saw that this nucleator appeared to move from the AJs to form the leading edges of cryptic lamellipodia^30^. This suggested that cryptic lamellipodia may derive from the dynamic actin cytoskeleton of AJs. Potentially, the WAVE-Arp2/3 nucleator complex is first recruited and activated at AJs before becoming incorporated into the lamellipodia. If so, AJs may not be necessary just to hold cells together as they are pulled during collective migration: they may help generate the organs of locomotion themselves.

How then might the cell regulate the fate to which junctional actin assembly is directed? Ozawa *et al*. provide an interesting lead. They showed that cryptic lamellipodia tend to be associated with conditions of increased contractile tension. Lamellipodia tended to appear preferentially at multicellular vertices, sites where contractile tension is concentrated^35,36^. Furthermore, cryptic lamellipodia were increased by deleting (knockout, or KO) the cadherin-associated protein α-catenin. α-catenin KO is expected to compromise cadherin adhesion. In experiments by Ozawa *et al*., it also increased the activity of myosin II, the principal contractile force generator in cells^30^. Furthermore, inhibiting contractility tended to restore locomotor abnormalities associated with α-catenin KO. Together, these observations suggested that increased cellular contractility could promote formation of cryptic lamellipodia.

Exactly how contractility encourages the assembly of lamellipodia from dynamic actin at AJs remains to be elucidated. One notion that we would suggest is that it may reflect the availability of space between cells^37^. The cell surfaces of neighbouring cells are not uniformly apposed to one another in coherent epithelia. Instead, they display intercellular spaces below the specialised apical junctional networks^38^. The separation of cell surfaces may be greater at multicellular vertices and might also be predicted to be increased when cell contractility is enhanced. This separation of cell–cell surfaces below the apical adhesions may then provide a route for actin assembly to support lamellipodia formation. In this model, newly assembled actin would be preferentially directed into the junctional cortex when cell surfaces are strongly apposed to one another but lamellipodia formation would be encouraged where subapical space is available. We would emphasise that the availability of subapical space is unlikely to be an independent variable. Instead, it may reflect a dynamic balance between forces that appose and separate adjacent surfaces. Indeed, Li *et al*. (2020) reported that, whereas contractility promoted separation, actin assembly promoted apposition of surfaces by generating microspikes that allowed cells to push upon one another^22^. Moreover, migrating cells have often been thought to have less stable junctions than non-migratory populations^15^.

Irrespective of the precise underlying mechanism, the link between AJs and cryptic lamellipodia is likely to be complex rather than all-or-nothing. Since E-cadherin can recruit the Arp2/3-WAVE apparatus, AJs in some form may be required for cryptic lamellipodia to be generated, but tuning adhesion or contractility (or both) can also influence their assembly. This potentially explains two interesting observations that link cell–cell interactions to collective migration. First, Ozawa *et al*. and another lab^39^ have found that epithelial cells grown in isolation were poorly migratory but became more motile when they formed clusters. One explanation for this finding is that AJs may provide the source of dynamic actin for cryptic lamellipodia. Second, the coordination of movement between cells is compromised when α-catenin is depleted in epithelial cells^40–42^. This was evident in α-catenin KO monolayers, where the ability of monolayers to close artificial wounds was compromised although the migration speed of individual constituent cells within the monolayers was not altered^30^. The reason for this discrepancy was that whereas wild-type cells tended to move directionally into the wounds, α-catenin KO cells frequently changed their direction of movement, resulting in a decrease in their net translocation. Here, what may be relevant is the number of lamellipodia that cells display. Directional movement is associated with the appearance of a small number of dominant protrusions^43^. Therefore, the increased number of cryptic lamellipodia that are seen in α-catenin KO cells may provide additional directional cues that misdirect cell migration.

## Junctional contraction and cell-on-cell movement

The studies that we have discussed so far treat cell–ECM adhesions as the major site where forces are applied to drive cell locomotion. However, cells can also exert forces on each other for cell-upon-cell locomotion^44^. This is best understood for so-called T1 transitions, as illustrated in Figure 1D, where we draw an epithelium as tiled hexagons. The individual junctions found between two cells are often called “bicellular” junctions, whereas those found at the vertices where multiple cells meet are known as “multicellular” (or “tricellular”) junctions. A T1 transition begins with the shortening and eventual loss of a bicellular junction (“Z” in Figure 1D), leading to a four-way vertex. This is resolved by the appearance of a new bicellular junction (“A”, Figure 1D) oriented approximately perpendicular (90°) to the first junction. Consequently, the cells that were originally apposed (Figure 1D) become separated (i.e., are moved) by the intercalation of their orthogonal neighbours. T1 transitions, and intercalations more generally^45^, underlie a range of morphogenetic movements in the embryo and in organs.

We therefore can think of junctional shortening as a locomotor apparatus for cell-on-cell migration, akin to the lamellipodia and cortical flows discussed above. Actomyosin-based contractility plays a key role in this junctional shortening that *initiates* the T1 transition. But how junctional shortening is regulated or constrained to influence cell movement varies in different biological contexts.

### Activating contractility for planar polarised intercalation

**Figure 2.**
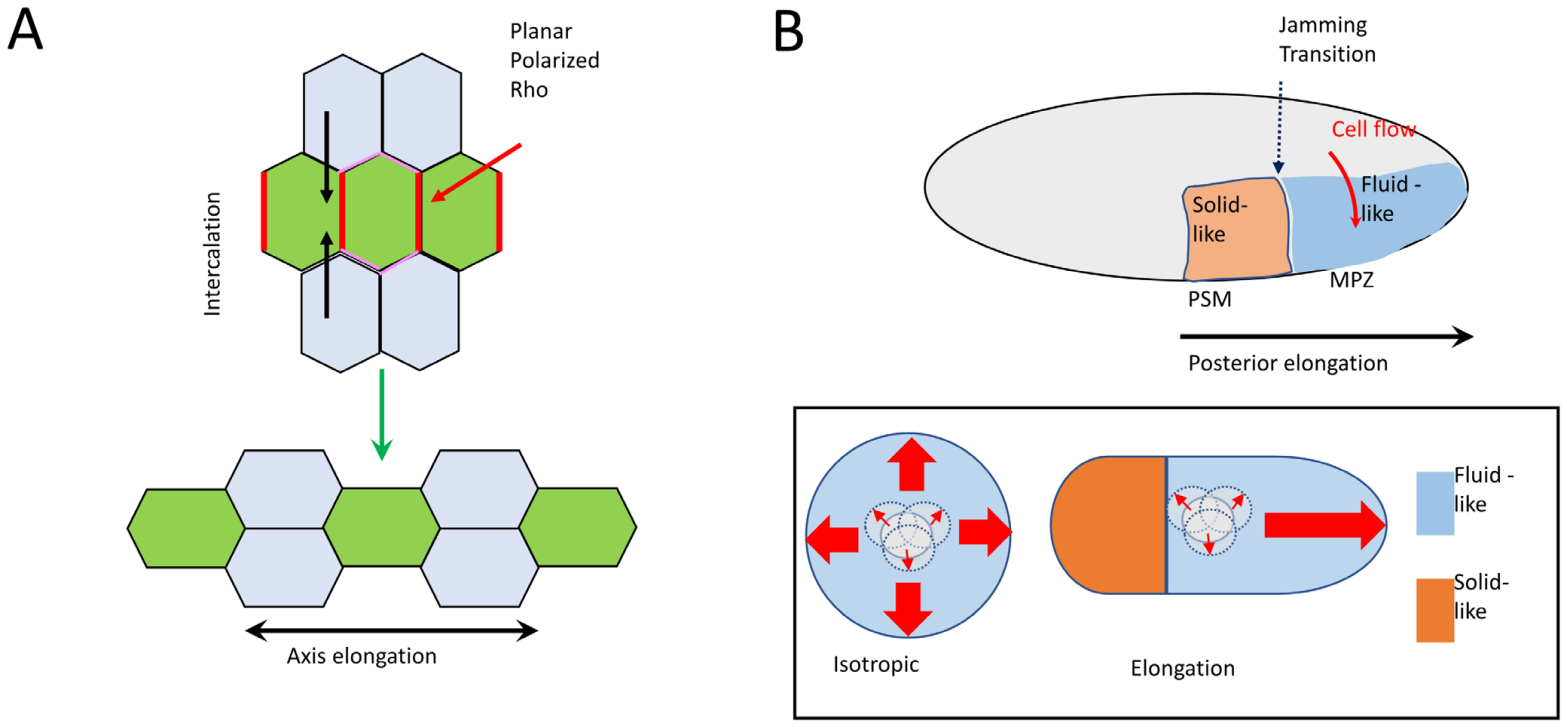
Embryo elongation: two different ways to harness junction contraction for morphogenesis. (**A**) In convergent extension, such as is associated with germband extension in *Drosophila*, cells undergo a planar polarised pattern of intercalation that leads to elongation of the body axis. In this case, intercalation is driven by preferential contraction of the vertical junctions, which are sites of elevated RhoA signalling. (**B**) Posterior elongation in the zebrafish embryo. Top: Here, cells move from the dorso-medial region of the embryo into the mesodermal progenitor zone (MPZ) and, upon differentiation, incorporate into the presomitic mesoderm (PSM). Stochastic junction contraction drives cell movement, but a jamming transition confers solid-like tissue properties on the PSM, which provides a solid foundation for the more fluid-like MPZ to elongate. Bottom (box): Conceptual schematic. One cell out of many shows a stochastic capacity for translocation (driven by contraction of the junctions to which it is connected). Where the whole tissue is fluid-like, this stochastic motility leads to isotropic expansion of the tissue (left). Where a region is solid-like (jammed), the more fluid-like region expands.

The first case that we will consider is exemplified by forms of convergent extension, such as germband extension in the *Drosophila* embryo^10,46^. Here, junctions contract in patterns that are planar-polarised in the tissue. In the example in Figure 2A, drawn from germband extension, junctions that are oriented in the dorso-ventral direction (also known as “vertical” junctions) shrink, ultimately leading to the horizontal intercalation of cells and extension of the body axis. Furthermore, junctions contract in a progressive, “ratchet-like” fashion^7^. This raises the question of how such spatial and temporal stereotypy can be brought about.

To date, much effort to understand planar polarised movement has focused on determining how actomyosin can be activated to contract a specific subset of cell–cell junctions. Two major pools of actomyosin have been implicated in junctional shortening: condensed networks found in the medial-apical cortex several microns away from the junctions and bundles found in close proximity to the cell–cell junctions themselves (Figure 1D). These pools often coexist; indeed, medial-apical actomyosin sometimes has been observed to flow toward the junctions as they contract^7^. These two pools of actomyosin make different contributions to junctional shortening in different contexts. For example, contraction of the medial-apical networks is principally responsible for apical constriction during tissue invagination^5^, junctional actomyosin appears to be the major driver of intercalations in the *Drosophila* notum^6^, while both the medial-apical and junctional pools contribute to germband extension in the fly^10^.

Both the junctional and medial-apical contractile networks are activated by RhoA (or Rho1 in *Drosophila*), which operates through downstream mediators, such as Rho kinase (ROCK) and formins. These mediators activate myosin and the assembly of F-actin bundles, respectively, that together facilitate contraction. But how is RhoA activity controlled in these different parts of the cortex? One answer to this problem lies in the upstream mechanisms that impinge on RhoA. RhoA is a guanine nucleotide-binding protein and its “activity” (i.e., its ability to bind and activate its downstream effectors) is determined by its nucleotide-bound status. GTP-loaded RhoA is active whereas GDP-RhoA is inactive. That nucleotide-loaded status is controlled, in turn, by a network of enzymes which promote GTP-loading (guanine nucleotide exchange factors, or GEFs) or promote the conversion of GTP to GDP (GTPase-activating proteins, or GAPs). Of note, a large number of GEFs exist and these, in particular, have long been hypothesised to control the spatial and temporal activation of RhoA^47^.

Recently, the Lecuit lab analysed this hypothesis in the context of *Drosophila* germband extension^10^. Here, active GTP-Rho1 is found with both the medial-apical and junctional pools of actomyosin that are required for intercalation^48^. Furthermore, junctional Rho1 is planar-polarised, being greater at vertical than horizontal junctions (Figure 2A), suggesting that this difference in its signal strength might contribute to the preferential shrinkage of those vertical junctions. One possibility is that these different pools of Rho1 are responding to different GEFs.

Indeed, De Las Bayonas *et al*. (2019)^10^ confirmed earlier reports that DRhoGEF2 was selectively required for medial-apical contractility. Disabling DRhoGEF2 by RNAi or a maternal/zygotic null mutant selectively depleted GTP-Rho1 from the medial-apical cortex but did not affect the junctional pool. The authors then identified a fly homologue of mammalian p114RhoGEF as being necessary to support junctional Rho1 signalling. Depletion of Dp114RhoGEF reduced active Rho1 at junctions and its overexpression increased junctional Rho1, but medial-apical Rho1 was not affected. Together, this suggested that DRhoGEF2 and Dp114RhoGEF were responsible for activating Rho1 in the medial-apical and junctional compartments, respectively.

These two GEFs, in turn, appear to respond to different upstream pathways, especially G protein–coupled receptor signalling. Earlier work had identified specific roles for the heterotrimeric G proteins Gα12/13 and Gβ13F/Gγ1 in regulating medial-apical and junctional contractility, respectively^49^. Indeed, Gα12/13 had been implicated in activating DRhoGEF2 for medial-apical contractility^49–51^. De Las Bayonas *et al*. further found that Dp114RhoGEF functioned downstream of Gβ13F/Gγ1. Strikingly, overexpression of Gβ13F/Gγ1 caused a planar polarised increase in GTP-RhoA at vertical junctions. Thus, the Gβ13F/Gγ1–Dp114RhoGEF pathway may play an important role in determining anisotropic patterns of Rho activation in this tissue to ultimately elongate the body axis.

### Antagonising contraction or constraining its impact or both

In addition to regulating contractile activation, collective migration can be influenced by processes which determine whether junctional contraction can be effectively translated into cell motion. We focus, in particular, on tissue tension and cellular jamming, which have recently emerged as factors that can constrain the locomotor impact of junctional contraction.

***1. Tissue tension and junctional shortening*.** So far, we have discussed how contractility may be stimulated to shorten bicellular junctions and induce cell intercalation. However, AJs often form a tensile network, especially within epithelia, that arises from the pre-existing coupling of adhesion to contractility. Could this pre-existing tension affect junctional shortening? If we consider the diagram in Figure 1D, junction Z must pull against the vertices at its ends if they are to shorten. As these vertices are points of intersection with other AJs, the pre-existing level of tension in the AJs could constitute a resistive load working against junction Z’s shortening. Moreover, although we draw four cells in Figure 1D, their AJs are part of the larger network in the tissue. In other words, the basal tension in the AJ *network* would antagonise junctional shortening. So, can the basal level of tension in AJs affect intercalations to influence motion in tissues?

Evidence for this concept was recently found in the *Drosophila* notum. Interestingly, intercalations in this tissue are driven by stochastic fluctuations in the contractility of individual bicellular junctions rather than by the planar polarised patterns seen in classic cases of convergent extension^6^. The basis for this stochasticity is not yet known but its contraction seems to involve the core contractile apparatus of the cytoskeleton. For example, the stochastic contraction of junctions in the notum was characteristically preceded by the local accumulation of myosin II. To analyse this further, Curran *et al*. (2017) developed a vertex model, whose parameters were informed by experimental data, to consider how junctional contractions embedded within a tensile network could influence tissue patterning^6^. The model confirmed that stochastic shortening of junctions would drive intercalations. Interestingly, it also predicted that intercalations would be antagonised by increasing tension in the network.

The authors then tested the prediction experimentally. They found that intercalations became more frequent when contractility was reduced by inhibiting ROCK and less frequent when contractility was increased by enhancing ROCK activity^6^. This was physiologically relevant as maturation of the notum was distinguished by a decrease in T1 transitions, along with an increase in junctional myosin II and tension. Therefore, tension in the junctional network can limit movement and intercalation by antagonising junctional shortening. Similarly, cell movements within cultured epithelial monolayers are reduced when junctional tension is increased^52^. Thus, tension in the epithelium, transmitted through the AJ network, may be a factor that constrains the ability of junctional contractility to drive cell-on-cell locomotion.

***2. Jamming transitions*.** Alternatively, the locomotor impact of junction contraction could be modulated if the movement of cells is caged, or their motion constrained, by the presence of neighbouring cells. This is the central intuition of “jamming”, a concept that was imported from the analysis of foams and colloids^53,54^. A jamming transition occurs when collectives of cells switch from showing fluid-like to solid-like behaviour (and the opposite applies to un-jamming transitions). The exact definition of a jamming transition influences how it is measured and this can vary from one study to another. One informative index is to consider the yield stress (i.e., the maximal mechanical stress than a tissue can sustain in a solid-like state before it begins to flow).

From this perspective, one might anticipate that a variety of biological features would influence the yield stress of a tissue. An obvious one is cell–cell adhesion which would be predicted to contribute positively to yield stress. Indeed, increased endocytosis of E-cadherin (i.e., decreased adhesion) was reported to induce an un-jamming transition in epithelial monolayers overexpressing Rab 5^13^. Similarly, evidence for un-jamming was seen when E-cadherin was depleted in mammary cancer cells^55^ or when N-cadherin was depleted from mesodermal precursors in the zebrafish embryo^8^. Understanding how reduced cell–cell adhesion can promote un-jamming, however, is influenced by the physical model used for analysis. Application of vertex models for epithelial mechanics has predicted roles for both cell–cell adhesion and cortical tension in promoting a jamming transition^54^. In contrast, Mongera *et al*. (2018) reported a role for the volume fraction of extracellular space during elongation of the zebrafish embryo^8^. Increasing the extracellular space, as occurred with depletion of N-cadherin, promoted un-jamming consistent with jamming scenarios in foams^54^.

Irrespective of the underlying physical basis for jamming, the more solid-like state of a “jammed” tissue would be expected to reduce local cell–cell rearrangements. Intuitively, this would be thought to reduce cell movement. However, Mongera *et al*. showed how jamming can be used to drive morphogenetic movement of a tissue. They studied posterior elongation in the Zebrafish embryo, a process where cells move from the dorsal-medial region of the embryo into the paraxial mesodermal progenitor zone (MPZ) (Figure 2B). Then, upon differentiation, the cells incorporate into the presomitic mesoderm (PSM). Interestingly, cell movement in this system appears to be also driven by stochastic fluctuations in junction length^8^, akin to what was described for the *Drosophila* notum. So, how can embryo elongation be achieved without the spatial regulation of junctional shortening that is seen with convergent extension?

The key appears to lie in spatial patterns of jamming and their attendant changes in tissue mechanics. Thus, Mongera *et al*. found that cells became progressively less motile as they move from the MPZ to the PSM, despite continuing to show stochastic contraction of their junctions (Figure 2B). This appeared to reflect increased jamming, which created zones with different mechanical properties within the embryo. The MPZ is fluid-like whereas the PSM is solid-like (Figure 2B). Interestingly, when the authors simulated morphogenesis computationally, they found that this jamming transition was necessary for axis elongation to occur: without jamming, the embryo enlarged isotropically. Incorporation of a localised jamming transition not only reproduced posterior elongation but also predicted vortices of cell movement, as have been observed experimentally. Thus, although the local effect of jamming is to decrease cell motion, jamming transitions can create morphogenetic movements at the tissue scale. In this instance, its jammed state would allow the PSM to create a rigid support that encourages more dynamic regions of the tissue to extend away from it (Figure 2B, conceptual model).

This emphasises that the collective pattern of motion in cell populations does not necessarily reflect the local patterns of movement by its constituent cells. This notion is illustrated by a study by Ilina *et al*. (2020), who examined the three-dimensional invasion of breast cancer cells into extracellular matrices^55^. Both naturally occurring cancers and experimental breast cancer models show a variety of patterns of invasion, ranging from migration as multicellular collectives to single-cell invasion. Ilina *et al*. found that E-cadherin promoted a collective phenotype when cells invaded into porous matrices and its depletion led to a pattern of single-cell invasion. Effectively, E-cadherin facilitated a jamming-like transition that supported collective migration when the ECM was porous. Interestingly, collective migration could be restored to E-cadherin–depleted cells by increasing matrix density to enhance tissue confinement, although the cells displayed evidence of an un-jammed state.

## Conclusions

Once upon a time, it was plausible to consider that AJs might principally contribute to collective migration by conferring mechanical integrity on moving cell populations. Those days are long past. It is now evident that AJs can contribute to different cellular processes that contribute to the many phenotypes of collective migration. We have focused on how AJs can participate in the force-generating apparatuses that allow cells to move and rearrange. We have also referred to how cell–cell interactions may constrain movement by jamming and transmitting tension. Cadherins can also influence other aspects of locomotility, such as promoting cell polarisation^8,56^ and focusing the cell protrusions needed for locomotion^43^. While we have principally considered epithelial models in our discussion, some of the lessons from these systems may apply in other tissues. For example, vascular endothelial (VE)-cadherin endocytosis also promotes collective migration of endothelia^57^, akin to the impact of E-cadherin endocytosis in epithelia^13^. It will be an interesting challenge to elucidate the molecular mechanisms that are responsible for these different effects of cadherins on cell locomotility. This is likely to involve mechanosensitive mechanisms that link different parts of the cells together. For example, mechanical tension has been reported to regulate the distribution of scaffolding proteins, such as Merlin, between AJs and cryptic lamellipodia^58^. It will also be important to understand how these cellular effects are coordinated to generate specific tissue-scale patterns of motion, from wound healing to tissue reshaping. Clearly, then, there is much left to learn.
